# An Image Preprocessing Model of Coal and Gangue in High Dust and Low Light Conditions Based on the Joint Enhancement Algorithm

**DOI:** 10.1155/2021/2436486

**Published:** 2021-11-12

**Authors:** Na Li, Xingyu Gong

**Affiliations:** College of Computer Science and Technology, Xi'an University of Science and Technology, Xi'an 710054, China

## Abstract

The lighting facilities are affected due to conditions of coal mine in high dust pollution, which bring problems of dim, shadow, or reflection to coal and gangue images, and make it difficult to identify coal and gangue from background. To solve these problems, a preprocessing model for low-quality images of coal and gangue is proposed based on a joint enhancement algorithm in this paper. Firstly, the characteristics of coal and gangue images are analyzed in detail, and the improvement ways are put forward. Secondly, the image preprocessing flow of coal and gangue is established based on local features. Finally, a joint image enhancement algorithm is proposed based on bilateral filtering. In experimental, *K*-means clustering segmentation is used to compare the segmentation results of different preprocessing methods with information entropy and structural similarity. Through the simulation experiments for six scenes, the results show that the proposed preprocessing model can effectively reduce noise, improve overall brightness and contrast, and enhance image details. At the same time, it has a better segmentation effect. All of these can provide a better basis for target recognition.

## 1. Introduction

**Figure 1:**
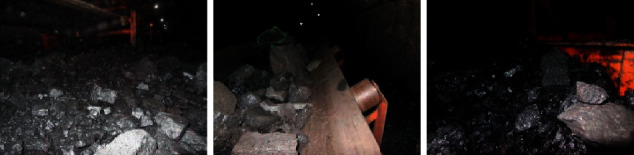
Coal and gangue images.

With development of information technology and application of artificial intelligence technology, coal mines are gradually moving towards the development of information and intelligence, such as unattended conveyor belt, intelligent mining of working face, and intelligent screening of coal. Coal and gangue recognition is an important technology in intelligent application of coal mine, and in intelligent process of coal and gangue recognition, visualization technology and image processing are important technical means. However, for the image obtained under special conditions of coal mine, it is affected by bad conditions, resulting in low illumination, noise, and other problems of the image, as shown in Figure 1. These low-quality images make it difficult to recognize coal and gangue, and also become a factor affecting the effect of coal and gangue recognition. Therefore, image preprocessing of coal and gangue is particularly important.

The feature extraction method based on gray and texture is the main research content for recognition and classification of coal and gangue images. In order to select the representative features of coal and gangue, the image preprocessing model in special conditions becomes the research content of this paper. Using smooth denoising and spatial domain enhancement technology in image processing, the image denoising and target enhancement methods are researched in high dust and low light conditions.

The rest of this article is organized as follows.  Section 2 is the analysis of low light image preprocessing technology.  Section 3 presents the preprocessing model for low-quality images of coal and gangue based on the joint enhancement algorithm.  Section 4 shows a contrast of different methods with our method, the model performance, and experimental results.  Section 5 presents the conclusions for our work.

## 2. Related Research on Technology of Low Light Image Preprocessing

In recent years, researchers have done a lot of work on low illumination image preprocessing [1]. It mainly includes algorithms based on histogram, defogging model based on atmospheric scattering, Retinex theory, and deep learning.

The key information such as the distribution of different gray levels can be well reflected by using the simplicity and effectiveness of histogram. Jiang et al. [2] combined the characteristics of global histogram equalization and local histogram equalization and used incremental histogram equalization to improve the histogram equalization algorithm. This kind of method can effectively remove the noise of low light image and enhance the overall brightness of image, but without selecting the processed data, so as to increase the contrast of useless signal and reduce the contrast of useful signal in background, which leads to the loss of local detail information and color distortion [3, 4].

In reference [5], the fog degradation model is used to enhance the low illumination image. Although the brightness of low illumination image is improved to a certain extent, the enhanced image is prone to noise and artifacts. In reference [6], the algorithm of enhancing low illumination image is proposed by the absorption light scattering model, which can reproduce the hidden contour and details from low illumination image and automatically adjust transmittance according to the image information. However, this method will make the original image with high brightness be overenhanced, resulting in loss of detail.

With the development of variational technology, image enhancement method based on Retinex theory has been widely concerned by researchers [7]. Retinex method has the characteristics of enhancing local contrast, compressing dynamic range, and maintaining image color constancy [8]. In reference [2], the maximum value of each pixel channel on the low illumination image is selected as the brightness map of image, and the regularization term is used to smoothen the brightness map, which achieves good results. However, the solving process involves Fourier transform and inverse Fourier transform, and the iterative method must be used to solve the problem, so the Fourier transform and inverse Fourier transform need to be repeated, which increases the complexity of the algorithm. The low illumination image enhancement algorithm based on Retinex theory has a good processing effect on the uneven illumination image, but the processing effect for the overall dark night image [9, 10] is not ideal, and it is also prone to halo phenomenon.

In low illumination image enhancement algorithm based on deep learning, it is necessary to collect and establish low illumination data sets for real scenes [11–14]. In the early days, due to lack of real data, the image is usually generated by researchers through some known methods such as random darkening and increasing noise, while the noise and image distortion of low illumination image in real scene are more complex. The dark light enhancement method based on the depth network [15] has some limitations [16], for example, LLNet based on self-coding is easy to lose its detail information in the process of image reconstruction, and RetinexNet network model does not consider the contour black edge effect and color distortion of the enhanced image, which will affect the visual effect of the image.

A variety of methods have also been applied to coal mine image preprocessing. According to the characteristics of coal mine image, a coal mine dust and fog image enhancement algorithm is proposed based on dark primary color theory and adaptive bilateral filtering in reference [17], which effectively enhance image details and edge information. The improved dust fog image clarity algorithm is proposed based on negative correction in reference [18], which effectively suppresses the halo effect. In reference [19], aiming at the problem of low illumination in main belt image of coal mine, a two-dimensional variational modal algorithm is proposed based on weighted guided filtering, and the image has good edge details. However, the algorithms for coal mine dust and fog images have some problems, such as overenhancement and lack of practicability.

In recent years, in order to improve the enhancement effect of low illumination image [20, 21], some methods are implemented by further adding constraints to histogram equalization. The visual effect of image is enhanced in these methods, and it also shows that there is more development space for low illumination image enhancement based on histogram equalization. In view of the shortcomings of image enhancement algorithm, such as artifacts, strong saturation, halo, and color distortion [22], how to adaptively enhance the low-quality image of coal and gangue in high dust and low light conditions still needs further research. In view of the defects of enhancement algorithm of low light image, a preprocessing model will be proposed for coal and gangue images with low-quality based on bilateral filtering and local histogram algorithm in this paper.

## 3. The Preprocessing Model for Low-Quality Images of Coal and Gangue

### 3.1. Characteristic Analysis of Coal and Gangue Images

Formation reasons of atomization image in coal and gangue recognition are mainly due to the small water droplets or ice crystals in the ambient air, as well as dust particles, which affect the color and clarity of images. In order to construct a low-quality image preprocessing model of coal and gangue in high dust and low light conditions, we analyze the characteristics of coal and gangue recognition image, and the details are as follows. (1) Due to the adverse environmental factors, various kinds of random noise (mainly salt and pepper noise and Gaussian white noise) are introduced in process of image conversion, image signal transmission, and signal processing, which lead to low image signal-to-noise ratio. (2) Due to the limitation of fixed acquisition location and single environment, the correlation between image pixels is large, which contains more redundant information. (3) The color information is weak, mainly black, white, and gray, and there is no large color discrimination, so the image information resources are limited. (4) The edge and detail information of coal and gangue are lost more. (5) The influence of fog caused by spray dust has increased the difficulty of image preprocessing.

In view of the above characteristics, the ways to improve and optimize the low-quality images in conditions of high dust and low light are as follows. (1) Replacing high-quality camera. (2) Reducing factors that affect the quality of video surveillance picture. (3) Improving quality of the video surveillance picture.

Simply improving camera level cannot achieve the purpose of shooting clear images in special conditions. In the bilateral filtering algorithm, the effect of noise is reduced by suppressing fog in images, but the result is dark and the speed of removing fog is slow, so we introduce gray histogram equalization algorithm to realize spatial enhancement according to local gray histogram characteristics of coal and gangue images.

### 3.2. Image Preprocessing Flow Based on Local Features of Coal and Gangue

**Figure 2:**
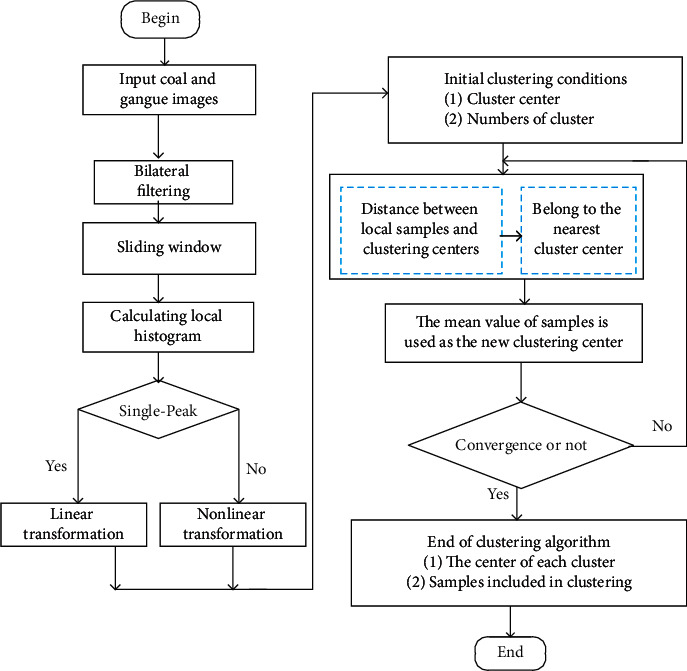
Flow chart of coal and gangue image preprocessing.

Through the analysis of local clustering features of coal and gangue images, the corresponding image preprocessing model is established, and the specific process flow is shown in Figure 2. According to the noise characteristics of coal and gangue, the spatial proximity and gray similarity are considered at the same time in the denoising part of image preprocessing. Using the nonlinear combination, bilateral filters are designed to smooth noise and preserve edge. In local neighborhood pixel processing, the gray transformation based on histogram is used to enhance contrast between the selected target and background. In order to preserve local features of original image effectively, a sliding window with fixed size is selected for local histogram processing. According to the single and double peaks of gray distribution, linear and nonlinear transformation is carried out to realize spatial enhancement based on the local gray histogram. Finally, the strong search ability of *K*-means clustering algorithm is used to achieve target segmentation.

### 3.3. A Joint Image Enhancement Algorithm Based on Bilateral Filtering

In bilateral filtering, the weighted average method is used, which not only considers the Euclidean distance of pixels but also considers the similarity in the range of pixels. These two weights are taken into account when calculating the center pixel. The operation of bilateral filtering is given in formulae (1) and (2), in which **M**_*q*_ is the input image and **M**_*p*_^*f*^ is the filtered image:

(1) $$\begin{array}{c} M_{p}^{f}=\frac{1}{W_{p}^{f}}\underset{q\in S}{\sum }G_{\sigma_{S}}\left(p-q\right)G_{\sigma_{r}}\left(M_{p}-M_{q}\right)M_{q}, \end{array}$$

(2) $$\begin{array}{c} W_{p}^{f}=\underset{q\in S}{\sum }G_{\sigma_{S}}\left(p-q\right)G_{\sigma_{r}}\left(M_{p}-M_{q}\right). \end{array}$$

In the above formula, *p*, *q*, (*p* − *q*) and (**M**_*p*_ − **M**_*q*_) represent the center point, current point, spatial distance, and pixel distance, respectively. The parameter *σ*_*S*_ is used to define the size of spatial neighborhood for filter pixels, and *σ*_*r*_ is used to control the degree of weight reduction for adjacent pixels due to the intensity difference. **W**_*p*_^*f*^ is the normalization factor to normalize the sum of weights. **G**_*σ*_*S*__ is the spatial weight, and **G**_*σ*_*r*__ is the gray range weight.

Spatial distance refers to the Euclidean distance between current point and center point in the filter template. At the same time, gray distance refers to the absolute value of difference between current point and center point in the filter template. The kernel function of bilateral filtering is the combination of spatial domain kernel and pixel domain kernel. In the flat area of the image, due to small change of pixel value, the weight of corresponding pixel domain is close to 1, so the weight of spatial domain plays a major role. At the same time, in the edge area of image, due to large change of the pixel value, the weight of the pixel domain becomes larger, so as to maintain the edge information. In bilateral filtering, the weight of pixel difference is used on the edge mutation, so the edge is well preserved.

In order to effectively maintain local features of coal and gangue image, the 5 *∗* 5 size of sliding window is selected to the filtered image **M**_*p*_^*f*^, and then the local histogram is processed. In this process, a neighborhood is defined and the center of region is moved from one pixel to another. At each location, the histogram of midpoint in domain is calculated to obtain histogram equalization transform function, which is finally used to map the gray level of center pixel in the neighborhood. Then, the domain center is moved to adjacent pixel position, and the process is repeated. The specific steps are as follows:

 
*Step 1*. Getting the histogram of first neighborhood
 
*Step 2*. Updating pixel of the center point of neighborhood according to histogram equalization
 
*Step 3*. Moving the center to the next neighborhood
 
*Step 4*. Executing Step 3 for all pixels

## 4. Simulation Experiment

### 4.1. Filtering for Coal and Gangue Images

**Figure 3:**
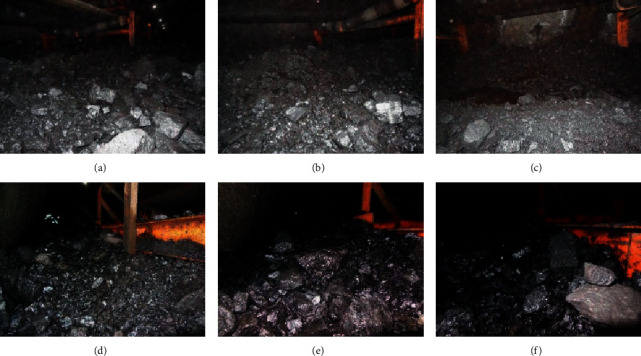
Scene images of coal mine. (a) Scene 1. (b) Scene 2. (c) Scene 3. (d) Scene 4. (e) Scene 5. (f) Scene 6.

In order to verify effectiveness of the method, we introduce six actual scene images, as shown in Figure 3.

**Figure 4:**
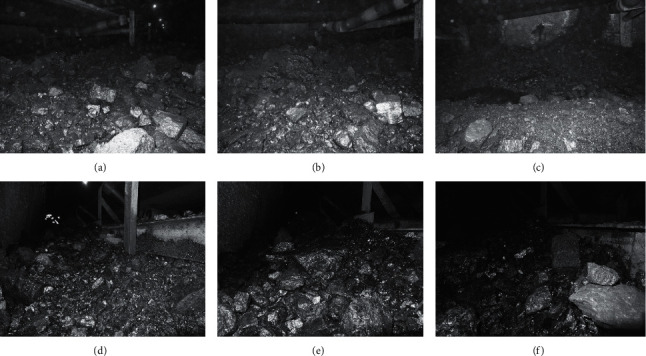
Bilateral filtering for scene images of coal mine. (a) Scene 1. (b) Scene 2. (c) Scene 3. (d) Scene 4. (e) Scene 5. (f) Scene 6.

In the experiment, the parameters of bilateral filter are filter radius *r*, global variance *a*, and local variance *b*. *r* is filter coefficients of geometric space distance, and *a* and *b* are filter coefficients of pixel. Here, we take *r* = 5, *a* = 3, and *b* = 0.01 for filtering. For six scene images, the bilateral filtering results are shown in Figure 4.

**Figure 5:**
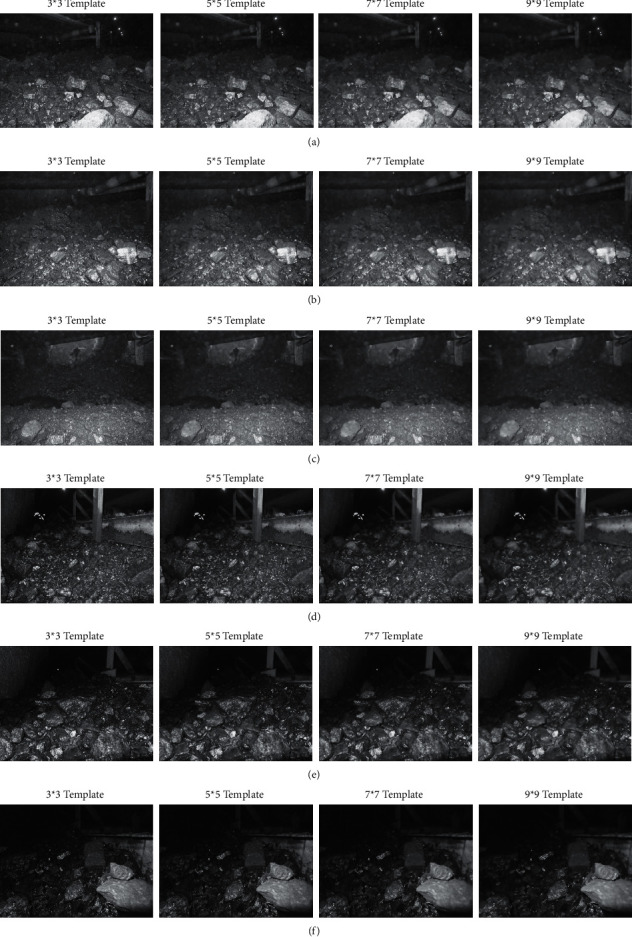
Adaptive filtering for scene images of coal mine. (a) Scene 1. (b) Scene 2. (c) Scene 3. (d) Scene 4. (e) Scene 5. (f) Scene 6.

In order to illustrate the image denoising effect of bilateral filtering, the bilateral filtering effect is compared with the adaptive filtering effect. Adaptive filter is used to filter the image pixel by pixel, and 3 *∗* 3, 5 *∗* 5, 7 *∗* 7, and 9 *∗* 9 templates are used to estimate the local mean and variance of the pixel. In order to simulate the image quality of coal mine conditions, Gaussian noise with mean value of 0 and variance of 0.002 is added. For six coal mine scene images, the adaptive filtering results are shown in Figure 5.

From the visual effect of the results in adaptive filtering experiment, there is a little noise in 3 *∗* 3 template, while 5 *∗* 5 template has a better effect, but in 7 *∗* 7 template and 9 *∗* 9 template, the target edge is smooth. In the adaptive filter, the local mean value and variance of each pixel in the image are estimated, and the adaptive way of pixel value is used for filtering, but the relationship between the spatial range of each pixel is not considered. Therefore, the edge of the target is fuzzy and the texture is not clear after filtering, which is particularly serious in 7 *∗* 7 and 9 *∗* 9 templates. It also makes the subsequent feature extraction difficult and increases the difficulty of coal and gangue recognition. However, in the bilateral filtering, the similarity degree in the pixel range domain, that is, the spatial distance, is considered. While filtering out the noise, the blur problem of the target edge and texture is improved, which provides rich local feature information for feature extraction.

In order to compare the two filtering results objectively, the adaptive filtering results of 5 *∗* 5 template and bilateral filtering results are taken. PSNR (peak signal-to-noise ratio) is introduced to evaluate the image quality after filtering. The higher the PSNR value is, the smaller the distortion is, that is, the better the quality is. The specific evaluation results are shown in Table 1.

For the same scene, the PSNR value of bilateral filtering is greater than that of adaptive filtering, which shows that the image quality of bilateral filtering is better for coal mine scene denoising.

### 4.2. Enhancement for Coal and Gangue Images

**Figure 6:**
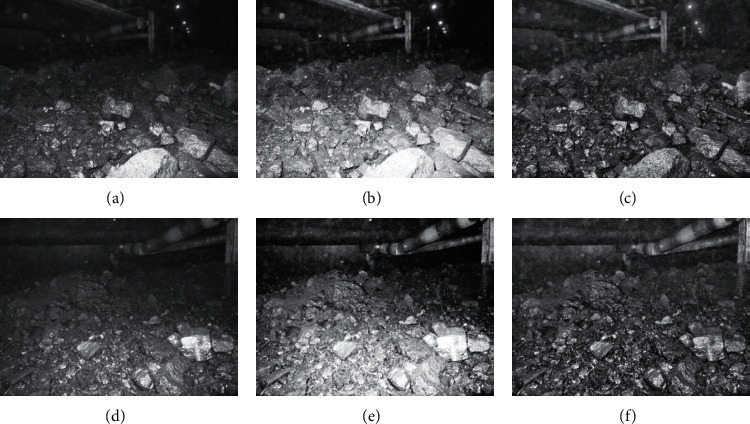
The enhancement of scene 1 and scene 2. (a) Scene 1. (b) The enhancement effect with global histogram. (c) The enhancement effect with local histogram. (d) Scene 2. (e) The enhancement effect with global histogram. (f) The enhancement effect with local histogram.

**Figure 7:**
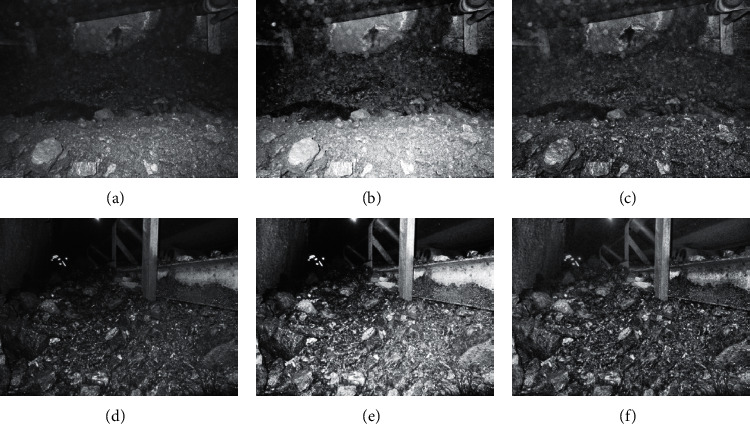
The enhancement of scene 3 and scene 4. (a) Scene 3. (b) The enhancement effect with global histogram. (c) The enhancement effect with local histogram. (d) Scene 4, (e) The enhancement effect with global histogram. (f) The enhancement effect with local histogram.

**Figure 8:**
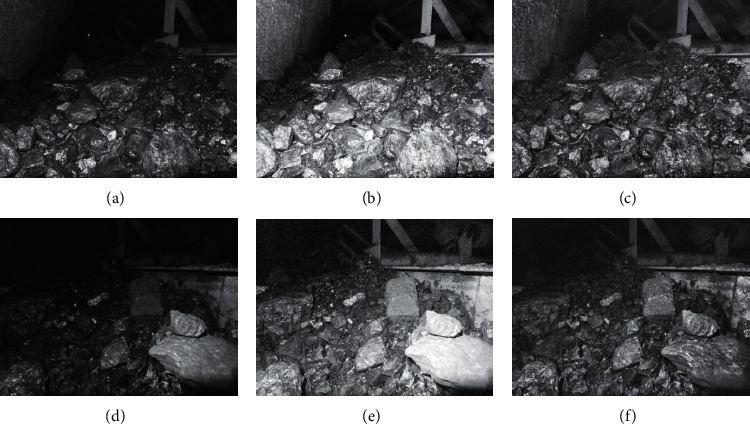
The enhancement of scene 5 and scene 6. (a) Scene 5. (b) The enhancement effect with global histogram. (c) The enhancement effect with local histogram. (d) Scene 6. (e) The enhancement effect with global histogram. (f) The enhancement effect with local histogram.

For the coal and gangue images after bilateral filtering, the global histogram and local histogram algorithm are, respectively, used for image enhancement processing, and the enhancement results are shown in Figures 6–8.

From the above three figures, we can see that, in global histogram and local histogram algorithm, the gray broadening is both achieved, that is, the effect of image enhancement. However, more uniform gray broadening is achieved in global histogram, while in local histogram algorithm, the gray change trend of original image is still retained in the process of image enhancement.

The histogram of coal and gangue images is equalized in the global histogram algorithm, but it cannot effectively maintain its local features, which is prone to color distortion. In the local histogram, the coal and gangue images are processed by the sliding window. The results show that the algorithm can effectively maintain the local features of original images and improve enhancement effect without obvious distortion.

In order to analyze the time complexity of the algorithm, we compare the running time of the image preprocessing algorithm based on bilateral filtering with that of the joint enhancement algorithm proposed in this paper. The average processing time of the former is 12.05 s for six actual scene images, and the average processing time of our algorithm is 12.18 s. It can be seen that the joint enhancement algorithm proposed in this paper achieves better enhancement effect for coal and gangue images without significantly increasing the computational overhead.

### 4.3. Objective Evaluation of the Experimental Results

In order to measure the role of the preprocessing model in coal and gangue segmentation, *K*-means clustering segmentation is performed on the adaptive filtering images, the bilateral filtering images, the enhanced images of global histogram, and the enhanced images of the method proposed in this paper, respectively, with six coal mine scenes. Then, we introduce information entropy and structural similarity (SSIM) to evaluate the results.

Let *p*_*i*_ represent the proportion of pixels with gray value *i* in the segmented image, then its information entropy is defined as follows:

(3) $$\begin{array}{c} H=-\sum_{i=0}^{255}p_{i}\log p_{i}. \end{array}$$

The clustering results are evaluated by the index of information entropy, as shown in Table 2. The larger the information entropy is, the better the segmentation effect is.

From the data in Table 2, it can be seen that the information entropy of *K*-means clustering segmentation of the scene images with bilateral filtering is greater than that of the adaptive filtering, which indicates that, for coal mine scene images, the bilateral filtering can retain more local information. Furthermore, on the basis of bilateral filtering, the global histogram enhancement and the local histogram enhancement are performed, respectively. The information entropy of the method proposed in this paper is the largest, which shows that the preprocessing model proposed in this paper can get better results for coal and gangue segmentation.

SSIM is used to measure the similarity of two images. The greater the value is, the more similar the structure of the two images is. The specific calculation formula is as follows:

(4) $$\begin{array}{c} \text{SSIM}\left(x,y\right)=\frac{\left(2\mu_{x}\mu_{y}+c_{1}\right)\left(2\mu_{xy}+c_{2}\right)}{\left(\mu_{x}^{2}+\mu_{y}^{2}+c_{1}\right)\left(\sigma_{x}^{2}+\sigma_{y}^{2}+c_{2}\right)}, \end{array}$$

where *x* and *y* are the two images for which the similarity needs to be calculated; *μ*_*x*_ is the mean value of *x*; *μ*_*y*_ is the mean of *y*; *σ*_*x*_^2^ is the variance of *x*; *σ*_*y*_^2^ is the variance of *y*; *σ*_*xy*_ is the covariance of *x* and *y*; and *c*_1_ and *c*_2_ are small disturbances.

The clustering results are evaluated by SSIM, as shown in Table 3. The better the clustering result is, the greater the difference from the original image structure is, that means the smaller the SSIM is, the better the segmentation effect is.

From the data in Table 3, it can be seen that the SSIM of the scene images with bilateral filtering is smaller than that of the adaptive filtering. The SSIM of the method proposed in this paper is the smallest, which also shows that the preprocessing model proposed in this paper can get better results for coal and gangue segmentation.

## 5. Conclusion

In this paper, according to characteristics of low-quality images of coal and gangue in conditions of high dust and low light, the corresponding image preprocessing model is established by summarizing existing problems in the process of coal and gangue recognition. The coal and gangue image enhancement algorithm is proposed based on bilateral filtering and local histogram. On this basis, *K*-means clustering segmentation is used to compare the segmentation results of different preprocessing methods. Through the simulation experiments for six scenes with different coal and gangue images, it shows that the method can effectively realize target image preprocessing for coal and gangue recognition.

## Figures and Tables

**Table 1:** PSNR comparison of bilateral filtering and adaptive filtering.

Methods	Bilateral filtering	Adaptive filtering
Scene 1	74.1705	73.8744
Scene 2	76.9725	75.5121
Scene 3	77.0361	75.9824
Scene 4	73.8625	71.4779
Scene 5	72.5784	70.7388
Scene 6	74.6327	73.0253

**Table 2:** Comparison of information entropy of different *K*-means clustering segmentation methods.

Methods	Adaptive filtering	Bilateral filtering	Global histogram	The method
Scene 1	2.2552	2.5855	2.8587	3.7505
Scene 2	2.2409	2.6583	3.3641	3.9814
Scene 3	1.8878	2.5136	3.2278	3.4684
Scene 4	5.1740	5.7263	5.7234	6.2987
Scene 5	3.8133	4.5667	4.8828	5.1010
Scene 6	2.0753	2.3148	3.2043	3.4844

**Table 3:** Comparison of SSIM of different *K*-means clustering segmentation methods.

Methods	Adaptive filtering	Bilateral filtering	Global histogram	The method
Scene 1	0.1976	0.1673	0.1406	0.0681
Scene 2	0.2614	0.2223	0.1623	0.0780
Scene 3	0.2903	0.2545	0.1580	0.1092
Scene 4	0.3001	0.2291	0.3543	0.0283
Scene 5	0.2457	0.1814	0.2662	0.0180
Scene 6	0.0856	0.0632	0.0542	0.0408

## Data Availability

The data used to support the findings of this study are available from the corresponding author upon request.
